# Systematic Review and Meta-Analysis of the Association between Ambient Nitrogen Dioxide and Respiratory Disease in China

**DOI:** 10.3390/ijerph14060646

**Published:** 2017-06-16

**Authors:** Jiyao Sun, Andrew J. Barnes, Dongyang He, Meng Wang, Jian Wang

**Affiliations:** 1 School of Health Care Management, Shandong University, Key Laboratory of Health Economics and Policy Research, National Health and Family Planning Commission of People’s Republic of China (NHFPC), Shandong University, 44 West Wenhua Road, Jinan 250012, China; sunjiyao108@163.com (J.S.); hedy_1993@126.com (D.H.); wangmenghlj@163.com (M.W.); 2 Department of Health Behavior and Policy, School of Medicine, Virginia Commonwealth University, 830 E Main St., Richmond, VA 23219, USA; andrew.barnes@vcuhealth.org

**Keywords:** nitrogen dioxide, respiratory disease, hospital admission, China

## Abstract

*Objective:* This study aimed to assess the quantitative effects of short-term exposure of ambient nitrogen dioxide (NO_2_) on respiratory disease (RD) mortality and RD hospital admission in China through systematic review and meta-analysis. *Methods:* A total of 29 publications were finally selected from searches in PubMed, Web of Science, CNKI and Wanfang databases. Generic inverse variance method was used to pool effect estimates. Pooled estimates were used to represent the increased risk of RD mortality and RD hospital admission per 10 μg/m^3^ increase in NO_2_ concentration. *Results:* Positive correlations were found between short-term NO_2_ exposure and RD in China. RD mortality and RD hospital admission respectively increased by 1.4% (95% CI: 1.1%, 1.7%) and 1.0% (95% CI: 0.5%, 1.5%) per 10 μg/m^3^ increase in NO_2_ concentration. Differences were observed across geographic regions of China. The risk of RD mortality due to NO_2_ was higher in the southern region (1.7%) than in the north (0.7%). *Conclusions*: Evidence was found that short-term exposure to NO_2_ was associated with an increased risk of RD mortality and RD hospital admission in China and these risks were more pronounced in the southern regions of the country, due in part to a larger proportion of elderly persons with increased susceptibility to NO_2_ in the population compared with the north.

## 1. Introduction

China has maintained a rapid rate of growth in its economy over the past three decades. However, the economic boom came the cost of worsening air quality [1]. Ambient air pollution, which is composed of both solid particles and gaseous pollutants, has been demonstrated to be associated with various adverse effects on human health [2]. Fuel combustion, automobile exhaust, industrial production has increased nitrogen dioxide (NO_2_) in the air [3]. Satellite data has indicated a significant increase of about 50% over the industrial areas of China from 1996 to 2004 [4]. Although many epidemiological studies have shown strong evidence linking air pollutants, such as particulates, NO_2_, sulfur dioxide (SO_2_), ozone with adverse health effects [3], it is still worth noting that NO_2_ which is a highly reactive, nitrogen-centred free radical, poorly water-soluble gas deposited peripherally in the lungs [5], probably more easily contribute to various respiratory diseases (RD), such as respiratory inflammation, responsiveness, infections and symptoms [6]. From a pathophysiological point of view, the main mechanism of NO_2_ toxicity has been suggested to involve lipid peroxidation in cell membranes and various actions of free radicals on structural and functional molecules [5], which could cause an airway inflammation, probably restricted to the smaller airways and the terminal bronchioles, at least after a single exposure [7].

More epidemiological evidence has also been established in recent years documenting that the NO_2_ concentration is increasing and is significantly correlated with increasing the risk of RD. As for mortality, Chen’s [3] study in 17 Chinese cities and Tao’s [8] study in the Pearl River Delta have estimated significant increases in RD mortality associated with short-term NO_2_ exposure. Wong [9] and Yang [10] also conducted studies from Hong Kong and Taipei that show consistent results. As for hospital admissions, Wong [11] and Zhang [12] investigated whether increases in NO_2_ concentration correspond to increases in RD hospital admissions in Hong Kong and Guangzhou. Tao [13] and Liu [14] also found positive associations between short-term NO_2_ exposure and RD hospital admissions in China.

With the increase of epidemiological studies, a systematic review and meta-analysis are needed to integrate the existing evidence on the correlation between NO_2_ and RD in Chinese population [15]. Some meta-analysis studies on the short-term adverse health effects of NO_2_ have been carried out in the United States [16,17], Canada [18] and Europe [5,19,20]. However, due to the difference of exposure level, air pollutant composition, and demographic characteristics between China and other countries, the exposure-response functions observed in other countries may not generalize to the Chinese context [21].

To the authors’ knowledge, quantitative systematic reviews and meta-analyses focusing on the association between NO_2_ and RD in China are limited. After a systematic database search, only three meta-analysis studies [22,23,24] were identified. These studies examined the epidemiological evidence prior to 2015, and only one of them showed a positive correlation between NO_2_ and RD hospital admission. Since 2015, the pace of epidemiological studies [25,26,27,28,29] published on the quantitative association between NO_2_ and RD has accelerated. However, no recent meta-analysis has been conducted to systematically review them. Therefore, the aim of the present study is to systematically collect and summarize the epidemiological evidence to date on RD mortality and RD hospital admission related to NO_2_ observed in the Chinese population published in either the English or Chinese peer-reviewed literature, in order to provide more up-to-date evidence to inform health impact assessment and air quality improvement.

## 2. Materials and Methods

### 2.1. Databases

We searched in the PubMed, Web of Science, China National Knowledge Infrastructure (CNKI) and Wanfang databases (last entry on 31 December 2016) for epidemiological literature on RD mortality and RD hospital admission due to ambient NO_2_ air pollution in China using the following terms: (1) nitrogen dioxide, NO_2_, air pollution; (2) respiratory disease, RD, disease, health, adverse effect, mortality, death, morbidity, hospital admission; and (3) China, Chinese, Taiwan, Hong Kong, river delta. The bibliographies of the articles identified using the search criteria above were also screened to expand our review. Furthermore, it is important to note that “respiratory disease (RD)” in present study referred to “all diseases of the respiratory system according to ICD-9 460-519 or ICD-10 J00-J98”, not including its single or sub-classification. EndNote Software (VersionX7, Thomson Corporation, Stamford, CT, USA) was employed to manage the citations.

### 2.2. Literature Selection and Data Extraction

All of the studies included in our review were selected using the following inclusion criteria: (1) Chinese and English epidemiological studies with health outcomes related to RD mortality and RD hospital admission due to short-term exposure to ambient NO_2_ in China; (2) studies focusing on short-term exposures, defined as the duration of up to 7 days to ambient NO_2_ associated with RD mortality and RD hospital admission; (3) articles included quantitative exposure-response associations (relative risk (RR), odds ratios (OR), excess risk (ER: also called attributable risk (AR), defined as the difference between the proportion of subjects in a population with a particular disease who were exposed to a specified risk factor and the proportion of subjects with that same disease who were not exposed) [30], and their 95% confidence intervals (95% CI)), and individual health risk estimates were expressed according to unit change in NO_2_ mass concentration; (4) study population was healthy people of all age and all gender; (5) exposure to ambient NO_2_ in the natural environment; (6) the analysis used the “International Classification of Diseases ICD-9 460-519 or ICD-10 J00-J98”; (7) time-series and case-crossover studies were used; (8) single-pollutant model results were available; and (9) for duplicated or derived articles conducted in same location, study period, data source, pollutants, or health outcomes, only the most recent publication was included. In addition, single-city study with different time periods from multi-cities study was also accepted [31]. The study followed the PRISMA guidelines for conducting systematic reviews and meta-analyses [32].

We excluded: (1) studies conducted in other countries; (2) studies presenting association between other air pollutants and other health outcomes; (3) reviews or duplicate publications; (4) conference abstracts without sufficient information, government reports, and other non-peer reviewed articles; (5) study duration less than one year; (6) studies with inconvertible data; (7) research without a city-specific quantitative short-term exposure-response relationship or only with stratified results; (8) studies with subjects designed to specific high risk groups (e.g., infants, children, the elderly, pregnant women, patients and smokers); (9) indoor, occupational or accidental exposures; (10) research on non-respiratory diseases, single or sub-classification of respiratory diseases.

For consistency, we generally ignored the distinctions between the measures of relative risk (RR) and odds ratio (OR) in present study, because the OR mathematically approximates the RR when the absolute risk of RD mortality and RD hospital admission due to NO_2_ exposure in population is low; we therefore extracted both of them in synthesis for simplicity [33]. Additionally, we followed Atkinson’s [34] protocols for selecting lags To be precise, if only one lag estimate was presented, it would be recorded for analyses; if multiple lags were presented, the lag would be selected based on the criteria: (1) the lag that the author focused on or stated as a priori; (2) the lag that was of the most statistically significance (positive or negative) and (3) the lag with the largest effect estimate (positive or negative). All individual effect estimates should be expressed as standardized increment in RD mortality and RD hospital admission due to per 10 μg/m^3^ change in NO_2_ concentration using the following formula:

(1) $${\mathrm{RR}}_{\left(\mathrm{standardized}\right)}={\mathrm{RR}}_{\left(\mathrm{original}\right)}^{\mathrm{Increment}\left(10\right)/\mathrm{Increment}\left(\mathrm{original}\right)}$$

1 ppb = 1.88 μg/m^3^ (1atmosphere and a temperature of 25 °C) (2)

(3) ER(%)=(RR−1)×100%

Two reviewers (Jiyao Sun and Dongyang He) independently screened the titles and abstracts. Then, full texts were reviewed to decide eligibility for inclusion. Disagreement was resolved by discussion. If the argument still existed, another reviewer (Meng Wang) was consulted to determine the final decisions. Data extraction was also conducted by two independent reviewers (Dongyang He and Meng Wang) for comparisons. Disagreements were resolved by consulting the third reviewer (Jiyao Sun).

In total, 29 studies were included in this meta-analysis. The title, authors, study period, publication year, study design, city, annual mean concentration of NO_2_, health risk estimates (RR or OR and 95% confidence interval), number of events and adjusted confounding factors were extracted and entered into Microsoft Excel database (Version 2010, Microsoft, Redmond, WA, USA). Stata Software (Version 12.0, StataCorp., College Station, TX, USA) was used to conduct meta-analysis.

### 2.3. Quality Assessment

Because no validated and standardized scales were recommended to assess the quality of time-series and case-crossover studies, we evaluated the quality of total 29 individual studies based on a scale from a related meta-analysis [35], which is composed of three items: respiratory disease (RD) diagnosis, air pollutant measurement and adjustment of confounders (Supplemental Table S1).

#### 2.3.1. RD Diagnosis

We determined the diagnosis of RD if coded according to International Classification of Diseases ICD-9 460-519 or ICD-10 J00-J98.

#### 2.3.2. Air Pollutant Measurement

We considered the study at low risk of bias if the frequency of air pollutant measurement was performed daily, otherwise high risk of bias would be recognized.

#### 2.3.3. Adjustment of Confounders

In terms of the potential confounders, long-term trends, seasonality, meteorological parameters (temperature and relative humidity), day of week (DOW), public holidays, influenza epidemics were recorded to assess the quality of the studies. Studies adjusting at least four items were considered at low risk of bias.

### 2.4. Meta-Analysis

In this meta-analysis, generic inverse variance method was used to summarize effects estimates from the individual studies identified [36]. We first examined the heterogeneity among all 29 studies using the standard I^2^ test. We observed heterogeneity at I^2^ exceeding 50%. We therefore used a random-effects model to pool effect estimates if I^2^ > 50% or otherwise used a fixed-effects model. We estimated heterogeneity through meta-regression and subgroup analysis. Then, funnel plots with Egger’s test were created to assess publication bias at an α level of 0.1 [37]. The trim-and-fill method [38] was used to adjust asymmetry if publication bias was observed. Finally, sensitivity analyses were performed to evaluate the robustness of the correlation results.

### 2.5. Sensitivity Analysis

To evaluate the robustness of the correlation results, we conducted a leave-one-out sensitivity analysis where the pooled meta-estimate was re-estimated excluding one study iteratively at a time to test the robustness of the main study findings to the exclusion of any single study [21]. If the pooled estimates remained stable after the sensitivity analysis, suggesting main results were not being driven by any single study.

## 3. Results

A total of 29 studies, including 14 Chinese studies and 15 English studies, from the Mainland China, Taiwan and Hong Kong were selected by searching in PubMed, Web of Science, CNKI and Wanfang databases and using the search criteria above (Supplemental Figure S1). These studies spanned research across 18 cities and included 25 time-series studies and four case-crossover studies. RD mortality and RD hospital admission were reported by 21 studies and eight studies, respectively (Table 1). All included studies were at low risk of bias according to the results of quality assessment. The annual mean concentrations of NO_2_ ranged from 23 to 83 μg/m^3^, and most of the reported concentrations exceeded the World Health Organization (WHO) air quality guidelines [39]. All RRs or ORs (95% confidence intervals) in the following contexts were based on a 10 μg/m^3^ increase in NO_2_ concentration.

The overall analyses have explained significant associations between ambient NO_2_ and RD in Chinese population in present meta-analysis, based on 29 studies [RD mortality: combined estimate (95% CI): 1.014 (1.011, 1.017); I^2^ = 65%; Egger’s test: *p* < 0.05; RD hospital admission: combined estimate (95% CI): 1.010 (1.005, 1.015); I^2^ = 66.2%; Egger’s test: *p* > 0.1].

### 3.1. Mortality

**Figure 1:**
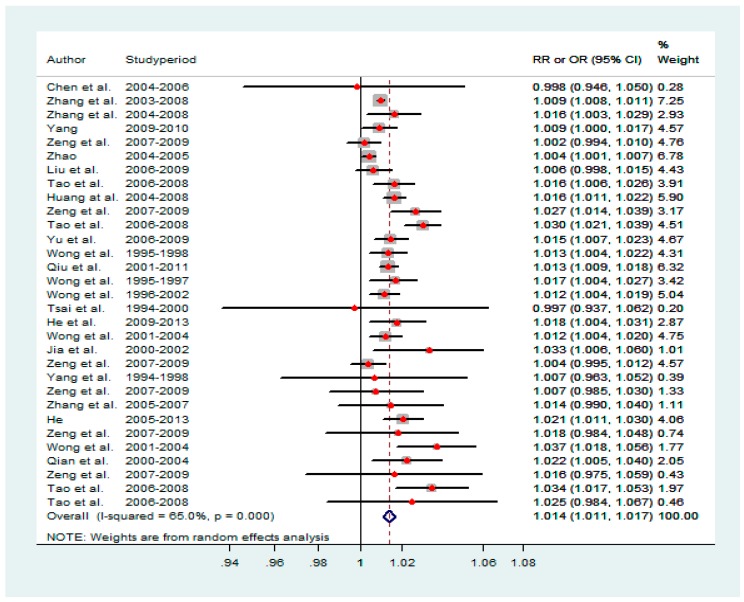
The effect of ambient NO_2_ pollution on RD mortality.

The effect estimates of RD mortality for each city by study are shown in Table 1. For each 10 μg/m^3^ increase in NO_2_ concentration, the risk of RD mortality increased by 1.4% (95% CI: 1.1%, 1.7%) (Figure 1). Significant heterogeneity was observed (I^2^ = 65%).

#### 3.1.1. Meta-Regression

For all of the selected studies, we conducted meta-regression by study geographical region, study design and annual mean concentration of NO_2_. Results showed that geographical region stratification (south and north) had a significant impact on the association between NO_2_ and RD mortality (*p* < 0.01). The effects of annual mean concentration of NO_2_ and study design (time-series and case-crossover) were not significant (*p* > 0.05) (Table 2).

#### 3.1.2. Subgroup Analysis

**Figure 2:**
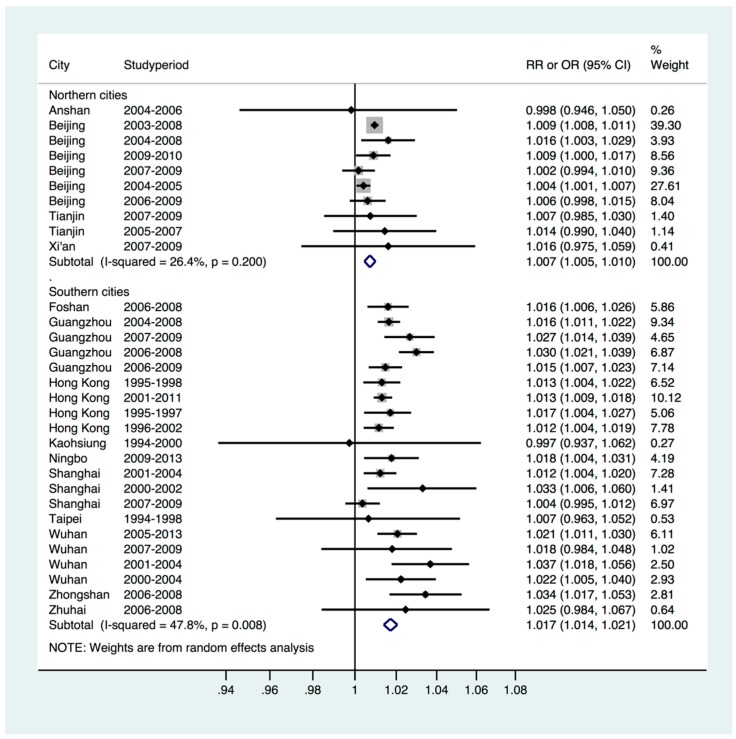
Regional analysis of the contribution of NO_2_ to RD mortality.

The main meta-regression models were stratified by geographic region in China. Results showed that heterogeneity significantly decreased after stratifying the model by region. For southern Chinese cities, each 10 µg/m^3^ increase in NO_2_ concentration was associated with an increased risk of RD mortality of 1.7% (95% CI: 1.4%, 2.1%), which was higher than the overall risk of 1.4%. For northern cities, each additional 10 µg/m^3^ increase in NO_2_ concentration was associated with an increased risk of RD mortality of 0.7% (95% CI: 0.5%, 1.0%), which was lower than the overall risk. Importantly, the increased risk of RD mortality associated with NO_2_ concentration in the southern area was higher than that in the north (Figure 2).

#### 3.1.3. Publication Bias and Sensitivity Analysis

Egger’s test showed a significant effect for publication bias (*p* < 0.05). Trim-and-fill method was used to adjust asymmetry, which did not alter the direction of the effect but reduced the effect size (adjusted overall combined estimates: 1.013; 95% CI: 1.010, 1.016).

The pooled estimate of RD mortality remained stable after the sensitivity analysis, suggesting main results were not being driven by any single study.

### 3.2. Hospital Admission

**Figure 3:**
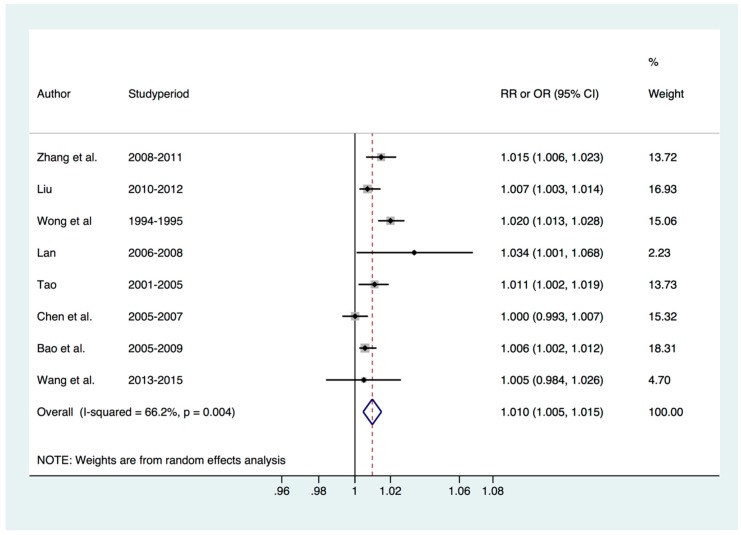
The effect of ambient NO_2_ pollution on RD hospital admission.

Hospital admissions were reported by 8 time-series studies in Jinchang, Lanzhou, Urumqi, Hong Kong, Guangzhou, Shanghai, Wuhan, Hangzhou. The estimates for each city by study are shown in Table 1. For each 10 μg/m^3^ increase in NO_2_ concentration, the risk of RD hospital admission increased by 1.0% (95% CI: 0.5%, 1.5%) (Figure 3).

#### 3.2.1. Heterogeneity and Sensitivity Analysis

Heterogeneity was detected across studies of RD hospital admission (I^2^ = 66.2%). Subgroup analysis by region and annual mean concentration of NO_2_ did not reduce the size of the heterogeneity estimate (Table 3). Furtherly, we found heterogeneity was significantly reduced by dropping Wong’s [12] study through sensitivity analysis. However, the combined effect size was reduced to 1.008 (95% CI: 1.003, 1.012) and was still positive (Table 4).

#### 3.2.2. Publication Bias

Egger’s test showed no significant publication bias effect was detected (*p* > 0.1).

## 4. Discussion

This study conducted a systematic review and meta-analyses of the evidence regarding the association between short-term exposure to NO_2_ and RD in China. In this quantitative analysis, the evidence suggests each 10 μg/m^3^ increase in NO_2_ concentration corresponded to 1.4% (95% CI: 1.1%, 1.7%) increase in RD mortality and 1.0% (95% CI: 0.5%, 1.5%) increase in RD hospital admission in the Chinese population. After stratifying by geographic region in China, we found the risk of RD mortality associated with NO_2_ concentration was higher in the southern area (1.7%) than in the north (0.7%).

The results found in this study can be compared with other studies conducted in other parts of the world. As for RD mortality, Lai’s meta-analysis [23] reported that in the Chinese population, a 10 μg/m^3^ increase in NO_2_ concentration was associated with a 2.2% increase in RD mortality. Their result was higher than that found in our study. The Public Health and Air Pollution in Asia (PAPA) [48] study showed the combined excess RD mortality risks was 1.63% per additional 10 μg/m^3^ of NO_2_ in three Chinese cities (Hong Kong, Shanghai, Wuhan). Chen [3] reported a combined estimate of 2.52% increase in RD mortality per 10 μg/m^3^ increase in NO_2_ in 17 Chinese cities. Shang’s [22] systematic review in China reported that an increase of 10 μg/m^3^ of NO_2_ was associated with a 1.62% increase in RD mortality. Atkinson [34] reported that a 10 μg/m^3^ increase of NO_2_ concentration corresponded to a 1.74% increases in RD mortality in a meta-analysis of time-series models from the Asian literature. Liu’s [24] meta-analysis reported a 10 μg/m^3^ increase in NO_2_ concentration corresponded to 1.39% increase in RD mortality in China. It was fair to suppose that the discrepancy was partially due to the changes among Chinese cities in levels of exposure, chemical compositions of ambient air pollution, city background characteristics such as urbanization level, medical and hygiene standards, age structure and underlying susceptibility of the population during the rapid economic development in recent years [23].

The European Approach (APHEA)-2 project included 30 European cities [5] and found that a 10 μg/m^3^ increase in NO_2_ concentrations was associated with a 0.38% increase in RD mortality, much lower than the association found in the current study. The EpiAir study [57] conducted in 10 Italian cities found statistically significant evidence that a 10 μg/m^3^ increase of NO_2_ was associated with a 3.48% increase in RD mortality, which was much higher than our result. It should be noted that Kinney and Özkaynak [58] found no significant association between respiratory deaths and NO_2_ concentration in Los Angeles County, USA. In terms of hospital admissions, only one meta-analysis study [23] reported a positive relationship between NO_2_ concentration and RD-associated hospital admission in Chinese population. Specifically, an increase of 10 μg/m^3^ of NO_2_ was associated with a 0.6% increase in RD hospital admission in the Chinese population. Chen’s [55] epidemiological research in Shanghai found that RD hospital admission was not significantly associated with NO_2_. However, Fusco’s [59] study in Italy found an increase in the interquartile range of NO_2_ concentration corresponded to 2.5% increase in RD hospital admissions. A quantitative summary of APHEA study results in five west European cities [60] revealed an almost 2% increase in RD hospital admission for an increase of 50 μg/m^3^ of NO_2_.

In our analysis of RD mortality, we found the risk associated with increased NO_2_ concentration in the southern areas of China was higher than that in the northern areas. Several reasons may explain these geographic differences. First, the differences in the age distribution of the populations residing in the northern and southern regions of China. Among the 10 provinces with the largest number of older adults in China, six are in the south [61]. Qian [62] reported that significant deterioration of respiratory function, respiratory infection, and even respiratory failure are more likely to occur in the elderly. Therefore, a higher percentage increase of RD mortality might happen in the southern area due to the preponderance of older adults residing there compared to the northern area. A second reason for these geographical differences may be due to variation in the sensitivity of the population to NO_2_ levels. For southern cities, the mean reported NO_2_ concentration is 51.9 μg/m^3^ whereas it is 53.7 μg/m^3^ for northern cities. Local residents in northern cities are living in a more serious NO_2_ polluted areas, and their respiratory system be more resistant to NO_2_ pollution [56]. This might cause lower excess risk of RD mortality in the north than in the south. In terms of hospital admissions, no significant difference was observed after stratification by geographic region. Additionally, we found that the heterogeneity observed was caused by one study (Wong) [11]. By comparing our results with other studies, we believed that study period, geographical features, climate patterns, pollutant composition and population sensitivity might lead to this heterogeneity [63].

What needs to be considered is that although the above results have indicated adverse effects of NO_2_ on RD mortality and RD hospital admission, but the role of NO_2_ as a surrogate of unmeasured pollutants cannot be ruled out [57]. Sarnat’s [64,65] research reported that high correlation between NO_2_ and PM_2.5_ have been found which suggest the possibility that the NO_2_ effects could be due in part to confounding from particulate matter through assessing epidemiological studies on adverse health effects of PM_2.5_, so NO_2_ may be considered mainly as a surrogate of ultrafine PM. Seaton and Dennekamp [66] consistently indicated that NO_2_ may serve as a marker for other pollutants, such as fine particles formed in high-temperature combustion of fossil fuels. Even though the existing multi-pollutant analyses are not competent for completely assessing the independent adverse health effects of NO_2_ which has the possibility as a surrogate of particulate matter, the present study did provide positive evidence that short-term exposure to NO_2_ was associated with an increased risk of RD mortality and RD hospital admission. Therefore, further research should be carried out to identify the independent effects of different pollutants, such as NO_2_ and their interaction [51].

## 5. Strengths

The current study has several notable strengths. First, we systematically collected and pooled additional epidemiological evidence that has been previously synthesized by expanding the publication year to 2016 in order to provide more current estimates of the magnitude of adverse health effects of ambient NO_2_ pollution in Chinese population. Second, we stratified the RD mortality estimates by geographical locations to provide contrasting result between southern area and northern area of China, and are the first find that risk of RD mortality associated with increasing NO_2_ concentration was higher in the southern area than in the northern area.

## 6. Limitations

Several potential limitations of the present analysis should be taken into account. First, publication bias was noted in analyses examining the relationship between NO_2_ and RD mortality. This may be due to the authors being more likely to emphasize and publish positive results than negative ones [22]. Second, heterogeneity existed in analyses of both RD outcomes perhaps because the individual study effects came from different study periods and from 18 different Chinese cities. The lag selection used might also have introduced heterogeneity into our meta-analysis [67]. Third, both time-series studies and case-crossover studies were selected in this meta-analysis. The results of case-crossover studies may be stronger than those of time-series studies, because case-crossover design can effectively control the confounding factors, such as smoking, diet, heredity, living conditions of the population and season factors by matching the case period and control period [68]. Thus, the inclusion of these two different study designs may have influenced the combined estimates and caused the heterogeneity we observed. Fourth, the combined estimates in present analysis may not accurately represent the actual exposure to NO_2_ to some extent because the exposure measurements used in each study were based on a small number of fixed outdoor monitors, a common limitation in ecological studies [69]. Furthermore, the age- and gender-specific data which were important effect modifiers for identifying subgroups susceptible to NO_2_ pollution exposure were also didn’t extracted in present study. Additionally, although all included studies have controlled the major confounders including long-term and seasonal trends, temperature and relative humidity, day of week, some other confounding factors, such as public hospitals and influenza epidemics, which have not been adjusted among all individual studies, may increase the risk of bias to a certain extent. Finally, only single-pollutant model results were included. We did not consider the potential synergistic effects of NO_2_ in multiple pollutants models [70].

## 7. Conclusions

The present meta-analysis provides updated scientific evidence that short-term exposure to ambient NO_2_ is associated increased risk of RD mortality and RD hospital admission in the Chinese population. Furthermore, extend the literature by being the first to find that the risk of RD mortality associated with increasing NO_2_ concentration in the southern area of China is higher than that in the northern area. Additional evidence is needed to guide Chinese policy makers to improve air quality and minimize the health impact of air pollutants.

## Figures and Tables

**Table 1:** RRs ^1^ or ORs ^2^ of RD ^3^ mortality and RD hospital admission due to short-term exposure to NO_2_^4^ and detailed information for each city by study.

Number	Author	Study Period	Publication Year	Study Design	No. of Events	City	Annual Mean Concentration of NO_2_ (μg/m^3^)	RR or OR and 95% CI ^5^			Adjusted Confounding Factors
								RR or OR	LCI	UCI	
**Mortality**											
[40]	Chen et al.	2004–2006	2010	Case-crossover	20,805	Anshan	25.5	0.9982	0.9461	1.0502	Long-term and seasonal trends, temperature, relative humidity, DOW ^6^.
[41]	Zhang et al.	2003–2008	2011	Time-series	4825	Beijing	64.8	1.00947	1.00759	1.01135	Long-term and seasonal trends, temperature, relative humidity, DOW, air pressure.
[42]	Zhang et al.	2004–2008	2011	Time-series	4165	Beijing	63.9	1.0161	1.003	1.0292	Long-term and seasonal trends, temperature, relative humidity, DOW.
[26]	Yang	2009–2010	2015	Time-series	15,038	Beijing	55.02	1.0089	1.0005	1.0172	Long-term and seasonal trends, temperature, relative humidity, DOW, air pressure.
[27]	Zeng et al. ^#^	2007–2009	2015	Time-series	13,505	Beijing	56.6	1.0017	0.9939	1.0096	Long-term and seasonal trends, temperature, relative humidity, DOW, public holidays.
[43]	Zhao	2004–2005	2007	Time-series	7241	Beijing	68.94	1.0041	1.0007	1.0074	Long-term and seasonal trends, temperature, relative humidity, DOW.
[44]	Liu et al.	2006–2009	2014	Time-series	17,532	Beijing	59.5	1.0061	0.9975	1.0148	Long-term and seasonal trends, temperature, relative humidity, DOW.
[8]	Tao et al.	2006–2008	2012	Time-series	5809	Foshan	70.4	1.016	1.006	1.0261	Long-term and seasonal trends, temperature, relative humidity, DOW, year, public holidays, influenza epidemics.
[45]	Huang et al.	2004–2008	2012	Time-series	27,740	Guangzhou	66.6	1.0164	1.011	1.0219	Long-term and seasonal trends, temperature, relative humidity, DOW.
[27]	Zeng et al.	2007–2009	2015	Time-series	6570	Guangzhou	60.1	1.0266	1.0142	1.039	Long-term and seasonal trends, temperature, relative humidity, DOW, public holidays.
[8]	Tao et al. ^#^	2006–2008	2012	Time-series	16,659	Guangzhou	53.9	1.0299	1.0213	1.0386	Long-term and seasonal trends, temperature, relative humidity, DOW, year, public holidays, influenza epidemics.
[46]	Yu et al.	2006–2009	2011	Time-series	8327	Guangzhou	47.69	1.0147	1.0066	1.0229	Long-term and seasonal trends, temperature, relative humidity, DOW.
[9]	Wong et al.	1995–1998	2009	Time-series	24,820	Hong Kong	56.4	1.013	1.004	1.022	Long-term and seasonal trends, temperature, relative humidity, DOW, influenza epidemics.
[28]	Qiu et al.	2001–2011	2015	Time-series	78,508	Hong Kong	-	1.013	1.009	1.018	Long-term and seasonal trends, temperature, relative humidity, DOW.
[47]	Wong et al.	1995–1997	2001	Time-series	18,732	Hong Kong	48.1	1.0171	1.0044	1.0273	Long-term and seasonal trends, temperature, relative humidity, DOW, public holidays, influenza epidemics.
[48]	Wong et al. ^#^	1996–2002	2008	Time-series	41,391	Hong Kong	58.7	1.0115	1.0042	1.0188	Long-term and seasonal trends, temperature, relative humidity, DOW, public holidays, influenza epidemics.
[49]	Tsai et al.	1994–2000	2003	Case-crossover	2811	Kaohsiung	53.79	0.9973	0.9365	1.0623	Long-term and seasonal trends, temperature, relative humidity, DOW.
[29]	He et al.	2009–2013	2016	Time-series	28,835	Ningbo	41.7	1.0177	1.0044	1.0311	Long-term and seasonal trends, temperature, relative humidity, DOW, air pressure and wind speed.
[48]	Wong et al.	2001–2004	2008	Time-series	20,893	Shanghai	66.6	1.0122	1.0042	1.0201	Long-term and seasonal trends, temperature, relative humidity, DOW, public holidays, influenza epidemics.
[50]	Jia et al.	2000–2002	2004	Case-crossover	1752	Shanghai	69.1	1.033	1.006	1.06	Long-term and seasonal trends, temperature, relative humidity, DOW, air pressure.
**Mortality**											
[27]	Zeng et al.	2007–2009	2015	Time-series	13,578	Shanghai	54.8	1.0036	0.9954	1.012	Long-term and seasonal trends, temperature, relative humidity, DOW ^6^, public holidays.
[10]	Yang et al.	1994–1998	2004	Case-crossover	3689	Taipei	57.45	1.0065	0.9628	1.0517	Long-term and seasonal trends, temperature, relative humidity, DOW.
[27]	Zeng et al.	2007–2009	2015	Time-series	6278	Tianjin	41.4	1.0074	0.9853	1.0301	Long-term and seasonal trends, temperature, relative humidity, DOW, public holidays.
[51]	Zhang et al.	2005–2007	2010	Time-series	4380	Tianjin	47	1.0144	0.9896	1.0398	Long-term and seasonal trends, temperature, relative humidity, DOW.
[52]	He	2005–2013	2014	Time-series	19,710	Wuhan	49.53	1.0206	1.0108	1.0302	Long-term and seasonal trends, temperature, relative humidity, DOW, public holidays.
[27]	Zeng et al.	2007–2009	2015	Time-series	1314	Wuhan	45.2	1.018	0.984	1.0476	Long-term and seasonal trends, temperature, relative humidity, DOW, public holidays.
[48]	Wong et al.	2001–2004	2008	Time-series	10,227	Wuhan	51.8	1.0368	1.0177	1.0563	Long-term and seasonal trends, temperature, relative humidity, DOW, public holidays, influenza epidemics.
[53]	Qian et al.	2000–2004	2007	Time-series	10,287	Wuhan	51.8	1.0223	1.0052	1.0396	Long-term and seasonal trends, temperature, relative humidity, DOW, year.
[27]	Zeng et al.	2007–2009	2015	Time-series	1898	Xi’an	54	1.0161	0.9746	1.0594	Long-term and seasonal trends, temperature, relative humidity, DOW, public holidays.
[8]	Tao et al.	2006–2008	2012	Time-series	4056	Zhongshan	48.4	1.0344	1.0167	1.0525	Long-term and seasonal trends, temperature, relative humidity, DOW, year, public holidays, influenza epidemics.
[8]	Tao et al.	2006–2008	2012	Time-series	1205	Zhuhai	38.1	1.0246	0.9841	1.0667	Long-term and seasonal trends, temperature, relative humidity, DOW, year, public holidays, influenza epidemics.
**Hospital admission**											
[12]	Zhang et al.	2008–2011	2014	Time-series	46,752	Guangzhou	56	1.0147	1.0062	1.0233	Long-term and seasonal trends, temperature, relative humidity, DOW, air pressure.
[14]	Liu	2010–2012	2014	Time-series	24,792	Hangzhou	83	1.007	1.0025	1.0144	Long-term and seasonal trends, temperature, relative humidity, DOW, public holidays.
[11]	Wong et al	1994–1995	1999	Time-series	61,320	Hong Kong	51.39	1.02	1.013	1.028	Long-term and seasonal trends, temperature, relative humidity, DOW, year, public holidays.
[54]	Lan	2006–2008	2012	Time-series	5808	Jinchang	23	1.0336	1.0009	1.068	Long-term and seasonal trends, temperature, relative humidity, DOW.
[13]	Tao	2001–2005	2009	Time-series	28,057	Lanzhou	46	1.011	1.002	1.019	Long-term and seasonal trends, temperature, relative humidity, DOW, public holidays.
[55]	Chen et al.	2005–2007	2010	Time-series	144,540	Shanghai	57	1.0001	0.993	1.0073	Long-term and seasonal trends, temperature, relative humidity, DOW, public holidays.
[56]	Bao et al.	2005–2009	2013	Time-series	24,935	Urumqi	48.8	1.0056	1.0024	1.0121	Long-term and seasonal trends, temperature, relative humidity, DOW.
[25]	Wang et al.	2013–2015	2016	Time-series	3314	Wuhan	63.13	1.005	0.984	1.026	Long-term and seasonal trends, temperature, relative humidity, DOW, public holidays, air pressure.

^1^ Relative risk; ^2^ Odds ratio; ^3^ Respiratory disease; ^4^ Nitrogen dioxide; ^5^ Confidence interval; ^6^ Day of week; ^#^ Multi-city studies including Zeng [27] Tao [8] and Wong [48].

**Table 2:** Results of meta-regression.

Category	Regression Coefficient	SE	T	P	95% CI	
					LCI	LCI
Region	1.009707	0.0026565	3.67	0.001	1.004289	1.015155
Concentration	0.999765	0.0001986	−1.18	0.247	0.9993583	1.000172
Study design	1.00385	0.010779	0.36	0.723	0.9820451	1.02614

**Table 3:** Subgroup analysis for the contribution of NO_2_ to RD hospital admission.

Stratification	Study Characteristics (Number of Studies)	Combined Estimate	95% CI		I^2^
			LCI	UCL	
Research region	Northern cities (3)	1.01	1.00	1.02	44.80%
	Southern cities (5)	1.01	1.00	1.02	75.70%
Annual mean concentration of NO_2_	23 μg/m^3^–53 μg/m^3^ (4)	1.01	1.00	1.02	75.00%
	53 μg/m^3^–83 μg/m^3^ (4)	1.01	1.00	1.01	55.00%

**Table 4:** Results of sensitivity analysis for the contribution of NO_2_ to RD hospital admission.

Study Omitted	Combined Estimates	95% CI		I^2^
		LCI	UCI	
Lan	1.009	1.004	1.015	65.7%
Tao	1.010	1.003	1.016	70.5%
Bao et al.	1.011	1.004	1.017	66.2%
Wong et al.	1.008	1.003	1.012	42.2%
Zhang et al.	1.009	1.003	1.015	67.6%
Chen et al.	1.011	1.006	1.016	58.4%
Wang et al.	1.010	1.004	1.016	70.8%
Lui	1.010	1.004	1.017	70.5%
Combined estimates	1.010	1.005	1.015	66.2%

## Supplementary Materials

The following are available online at www.mdpi.com/1660-4601/14/6/646/s1, Figure S1: Flow chart for study retrieval and selection process, Table S1: Quality assessment of included studies in the systematic review.
