# Supplementary material for: Systematic Review and Meta-Analysis of the Association between Ambient Nitrogen Dioxide and Respiratory Disease in China
Source: Int J Environ Res Public Health. 2017 Jun 16;14(6):646. doi: 10.3390/ijerph14060646 (PMC5486332; doi:10.3390/ijerph14060646)
Supplement: Supplementary file 1 [file ijerph-14-00646-s001.pdf]

Systematic review and meta-analysis of the association between ambient nitrogen dioxide and respiratory disease in China

Figure S1. Flow chart for study retrieval and selection process

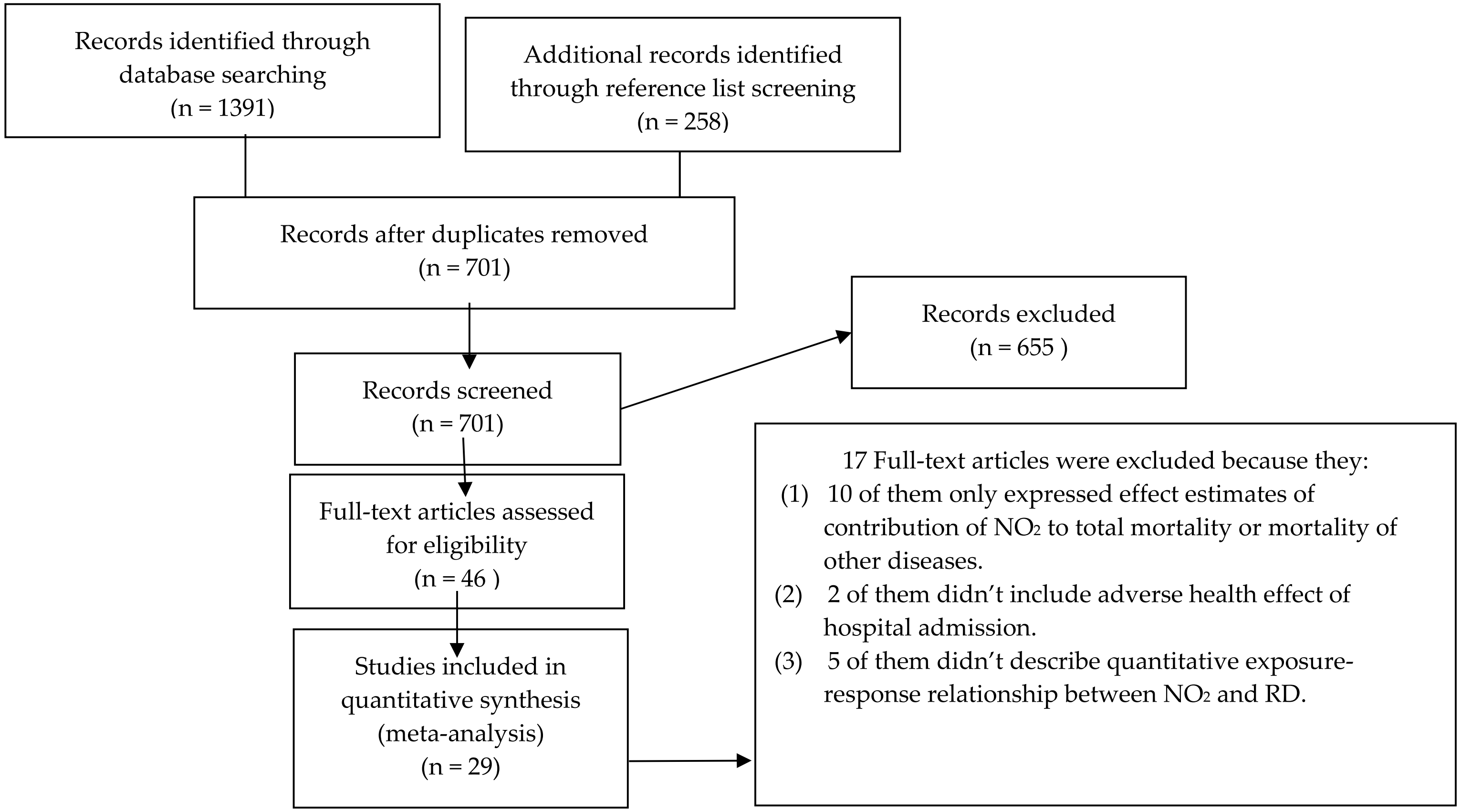

Table S1 Quality assessment of included studies in the systematic review

Number	Author	Diagnostic Evidence of Disease	Daily Pollutant Measurement	Long-term Trends	Seasonality	Meteorological Parameters [1]	DOW [2]	Public Holidays	Influenza Epidemics	Risk of Bias
**Mortality**										
[32]	Chen et	ICD-10 [5]	Y [3]	Y	Y	Y	Y	N [4]	N	Low
[33]	Zhang et	ICD-10	Y	Y	Y	Y	Y	N	N	Low
[34]	Zhang et	ICD-10	Y	Y	Y	Y	Y	N	N	Low
[22]	Yang	ICD-10	Y	Y	Y	Y	Y	N	N	Low
[23]	Zeng et	ICD-10	Y	Y	Y	Y	Y	Y	N	Low
[35]	Zhao	ICD-10	Y	Y	Y	Y	Y	N	N	Low
[36]	Liu et al.	ICD-10	Y	Y	Y	Y	Y	N	N	Low
[3]	Tao et al.	ICD-10	Y	Y	Y	Y	Y	Y	Y	Low
[37]	Huang et	ICD-10	Y	Y	Y	Y	Y	N	N	Low
[23]	Zeng et	ICD-10	Y	Y	Y	Y	Y	Y	N	Low
[3]	Tao et al.	ICD-10	Y	Y	Y	Y	Y	Y	Y	Low
[38]	Yu et al.	ICD-10	Y	Y	Y	Y	Y	N	N	Low
[4]	Wong et	ICD-9	Y	Y	Y	Y	Y	N	Y	Low
[24]	Qiu et al.	ICD-10	Y	Y	Y	Y	Y	N	N	Low
[39]	Wong et	ICD-9	Y	Y	Y	Y	Y	Y	Y	Low
[40]	Wong et	ICD-9, ICD-	Y	Y	Y	Y	Y	Y	Y	Low
[41]	Tsai et al.	ICD-9	Y	Y	Y	Y	Y	N	N	Low
[25]	He et al.	ICD-10	Y	Y	Y	Y	Y	N	N	Low
[40]	Wong et	ICD-9, ICD-	Y	Y	Y	Y	Y	Y	Y	Low
[42]	Jia et al.	ICD-9, ICD-	Y	Y	Y	Y	Y	N	N	Low

[1] Meteorological Parameters: Refers to temperature and relative humidity in our study; [2] Day of week; [3] Yes; [4] No; [5] International Classification of Diseases

Table S1 Quality assessment of included studies in the systematic review (continued)

Number	Author	Diagnostic Evidence of Disease	Daily Pollutant Measurement	Long-term Trends	Seasonality	Meteorological Parameters [1]	DOW [2]	Public Holidays	Influenza Epidemics	Risk of Bias
**Mortality**										
[23]	Zeng et	ICD-10 [5]	Y [3]	Y	Y	Y	Y	Y	N [4]	Low
[5]	Yang et	ICD-9	Y	Y	Y	Y	Y	N	N	Low
[23]	Zeng et	ICD-10	Y	Y	Y	Y	Y	Y	N	Low
[43]	Zhang	ICD-10	Y	Y	Y	Y	Y	N	N	Low
[44]	He	ICD-10	Y	Y	Y	Y	Y	Y	N	Low
[23]	Zeng et	ICD-10	Y	Y	Y	Y	Y	Y	N	Low
[40]	Wong et	ICD-9,	Y	Y	Y	Y	Y	Y	Y	Low
[45]	Qian et	ICD-9,	Y	Y	Y	Y	Y	N	N	Low
[23]	Zeng et	ICD-10	Y	Y	Y	Y	Y	Y	N	Low
[3]	Tao et	ICD-10	Y	Y	Y	Y	Y	Y	Y	Low
[3]	Tao et	ICD-10	Y	Y	Y	Y	Y	Y	Y	Low
**Hospital**										
[7]	Zhang	ICD-10	Y	Y	Y	Y	Y	N	N	Low
[9]	Liu	ICD-10	Y	Y	Y	Y	Y	Y	N	Low
[6]	Wong et	ICD-9	Y	Y	Y	Y	Y	Y	N	Low
[46]	Lan	ICD-10	Y	Y	Y	Y	Y	N	N	Low
[8]	Tao	ICD-10	Y	Y	Y	Y	Y	Y	N	Low
[47]	Chen et	ICD-10	Y	Y	Y	Y	Y	Y	N	Low
[48]	Bao et	ICD-9,	Y	Y	Y	Y	Y	N	N	Low
[21]	Wang et	ICD-10	Y	Y	Y	Y	Y	Y	N	Low

[1] Meteorological Parameters: Refers to temperature and relative humidity in our study; [2] Day of week; [3] Yes; [4] No; [5] International Classification of Diseases.
